# Myocardial Scar Characterization and Future Ventricular Arrhythmia in Patients With Ischemic Cardiomyopathy and an Implantable Cardioverter-Defibrillator

**DOI:** 10.3389/fcvm.2021.708406

**Published:** 2021-08-17

**Authors:** Alwin B. P. Noordman, Alexander H. Maass, Hessel Groenveld, Bart A. Mulder, Michiel Rienstra, Yuri Blaauw

**Affiliations:** Department of Cardiology, Heart Center, University of Groningen, University Medical Center Groningen, Groningen, Netherlands

**Keywords:** ventricular arrhythmia, implantable cardioverter-defibrillator, ischemic cardiomyopathy, myocardial scar, late gadolinium enhancement

## Abstract

**Background:** Implantable cardioverter-defibrillator (ICD) therapy is associated with several deleterious effects, which can be reduced by antiarrhythmic drugs or catheter ablation. However, it is largely unknown which patients might benefit from these therapies. Therefore, this study aimed to investigate whether myocardial scar characterization improves risk stratification for ventricular arrhythmia (VA) occurrence in patients with ischemic cardiomyopathy and an ICD.

**Methods:** In this study, 82 patients with ischemic cardiomyopathy who received an ICD were enrolled retrospectively. Late gadolinium enhancement cardiac magnetic resonance (LGE-CMR) images were analyzed using an investigational software tool to obtain quantitative data regarding the total scar, core, and border zone (BZ). Data regarding the QRS complex was obtained from electrocardiography (ECG). The primary endpoint was appropriate ICD therapy.

**Results:** During a median follow-up duration of 3.98 years [interquartile range (IQR) 2.89–5.14 years], appropriate therapy occurred in 24 (29.3%) patients. Patients with appropriate ICD therapy had a significantly larger total scar mass [60.0 (IQR 41.2–73.4) vs. 43.3 (IQR 31.2–61.2) g; *P* = 0.009] and BZ mass [32.9 (IQR 26.9–42.4) vs. 24.5 (IQR 18.8–32.5) g; *P* = 0.001] than those without appropriate therapy. In multivariable Cox regression analyses, total scar mass [hazard ratio (HR) 1.02 [95% confidence interval (CI) 1.00–1.04]; *P* = 0.014] and BZ mass (HR 1.04 [95% CI 1.01–1.07]; *P* = 0.009) independently predicted appropriate ICD therapy. Core mass and the QRS complex, however, were not significantly associated with the primary endpoint.

**Conclusion:** LGE-CMR-based, but not ECG-based myocardial scar characterization improves risk stratification for VA occurrence in patients with ischemic cardiomyopathy who received an ICD.

## Introduction

Sudden cardiac death (SCD), which is defined as an unexpected and non-traumatic death from a cardiovascular cause, is a leading cause of mortality, being responsible for a fifth of all deaths worldwide (1, 2). The most common underlying etiology is coronary artery disease, because of the arrhythmogenicity of post-infarction myocardial scar tissue (1). Both acute myocardial ischemia and scar tissue from a previous myocardial infarction can lead to SCD (3). An implantable cardioverter-defibrillator (ICD) can be implanted both for primary and secondary prevention of SCD in patients at high risk. In response to the detection of ventricular arrhythmias (VAs) by the ICD, which include ventricular fibrillation (VF) and ventricular tachycardia (VT), patients receive appropriate ICD therapy, including ICD shocks (4). Although these devices are effective in reducing mortality (4), both ICD shocks and antitachycardia pacing (ATP) are associated with anxiety (5), and may have deleterious psychological effects (6). These negative effects can be reduced by preventing the occurrence of VT with therapies such as antiarrhythmic drugs and catheter ablation (6).

Several predictors of appropriate ICD shock have been found, including male gender, non-sustained VT, and a history of atrial fibrillation (7). Wide QRS and VT as presenting arrhythmia are other examples of predictors of VAs in secondary prevention ICD recipients (8). The presence of myocardial scar tissue can be identified by late gadolinium enhancement cardiac magnetic resonance (LGE-CMR). The characterization and quantification of myocardial scars by LGE-CMR, including their core and border zone (BZ), has proven useful for the risk stratification of VAs. Specifically, the BZ has been found to be important for the prediction of the occurrence of VAs and subsequent appropriate ICD therapy (9–14). The presence of a myocardial scar can also be inferred from electrocardiography (ECG), which is commonly used in clinical practice and constitutes a more feasible method of large-scale population screening than LGE-CMR (15). Several electrocardiographic markers indicating the presence of myocardial fibrosis have been found, including pathological Q waves (15). In addition, it has been demonstrated that QRS fragmentation, which is a predictor of mortality and SCD, is associated with myocardial fibrosis (15, 16).

There is a need for more studies investigating the value of myocardial scar characterization for the prediction of the occurrence of VAs. Similarly, it is largely unknown which patients are at high risk of VAs and therefore might benefit from antiarrhythmic drugs or VT ablation. Since the prevention of the occurrence of VAs by these therapeutic methods can limit the deleterious effects of ICD therapy and improve the quality of life of ICD recipients, the identification of patients at high risk of arrhythmias, and thus ICD therapy is important. By improving risk stratification for VA occurrence, myocardial scar characterization could facilitate appropriate clinical decision making.

To determine which patients have an increased risk of arrhythmias and therefore might benefit from antiarrhythmic drugs or catheter ablation, we investigated whether myocardial scar characterization improves risk stratification for VA occurrence in patients with ischemic cardiomyopathy and an ICD.

## Materials and Methods

### Patient Population

**Figure 1:**
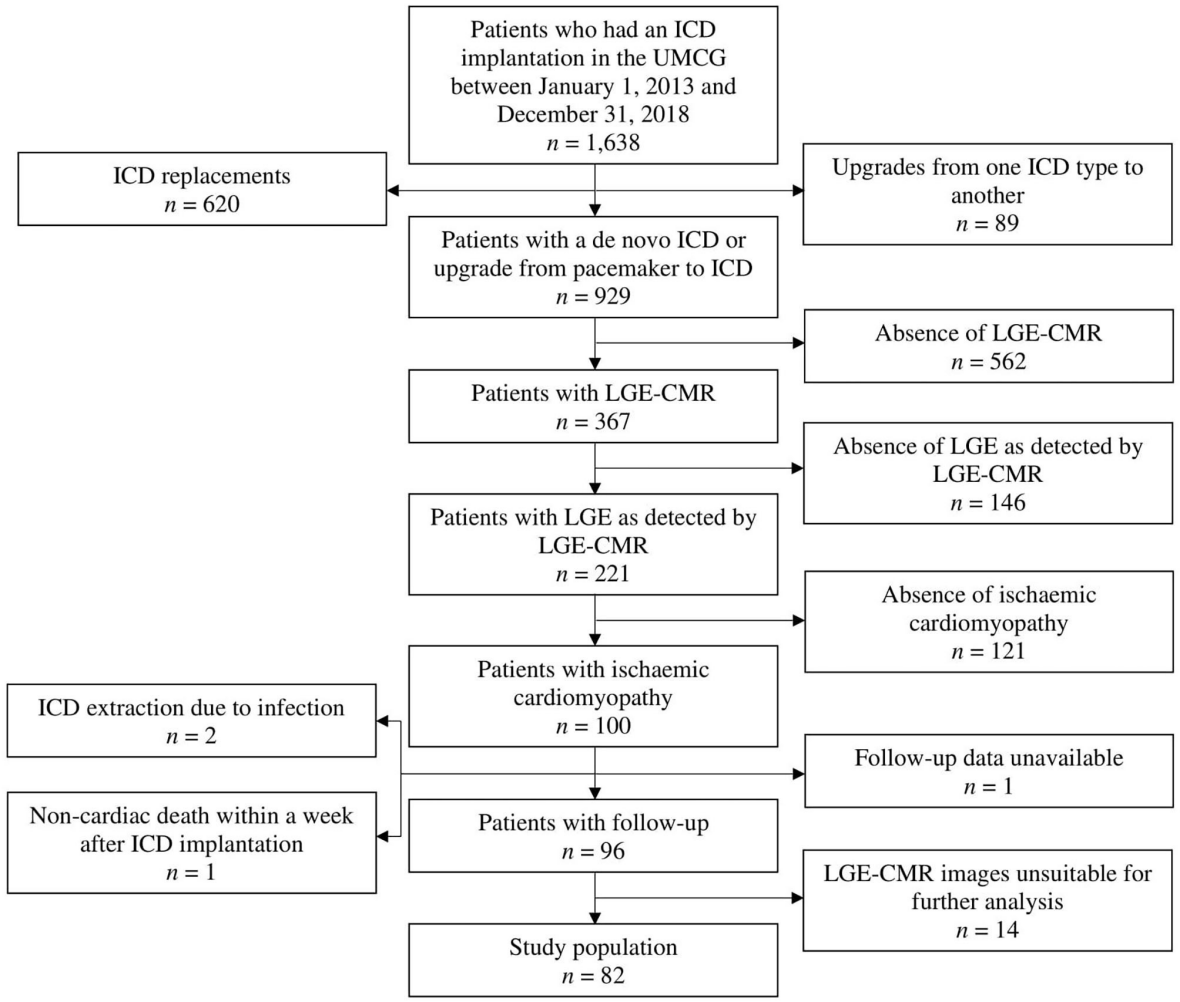
Flow diagram displaying the applied patient selection procedure. ICD, implantable cardioverter-defibrillator; LGE, late gadolinium enhancement; LGE-CMR, late gadolinium enhancement cardiac magnetic resonance; UMCG, University Medical Center Groningen.

In this single-center observational study, 82 patients with ischemic cardiomyopathy who received an ICD for primary or secondary prevention indications were enrolled retrospectively. All patients received their ICD in the University Medical Center Groningen (UMCG) in the period between January 1, 2013 and December 31, 2018. All patients had a previous history of myocardial infarction. Patients were included if they met all of the following criteria: a *de novo* ICD implantation or upgrade from pacemaker to ICD, the fact that LGE-CMR imaging was performed, the presence of a myocardial scar as detected by LGE-CMR, the presence of an ECG, and age ≥18 years. Patients with an ICD replacement or an upgrade of their ICD to another type and patients with an underlying pathology other than ischemic cardiomyopathy were excluded from further analysis. Patients were also excluded if follow-up data was lacking for any of the following reasons: ICD extraction soon after its implantation due to infection, non-cardiac death within a week after ICD implantation, or unavailability of follow-up data. Finally, patients whose LGE-CMR images were unsuitable for further analysis (*n* = 14) were excluded as well (Figure 1).

At the time of this study, the use of anonymous data was allowed by the Dutch Central Committee on Research Involving Human Subjects (CCMO) without the requirement of obtaining prior approval of an Institutional Review Board, with the provision that the data are acquired for routine patient care. All data used were handled anonymously. This study was conducted in compliance with the Declaration of Helsinki.

### Data Acquisition

Primary prevention was defined as an ICD implantation for symptomatic heart failure (HF) and a left ventricular ejection fraction (LVEF) ≤ 35%. A documented VF or sustained VT with hemodynamic compromise prior to the implantation of an ICD defined secondary prevention (17). Electrocardiography data with regard to the QRS complex, which was obtained prior to ICD implantation, was categorized as normal QRS complex, broad QRS complex, notched QRS complex, and fragmented QRS complex. Fragmented QRS was defined by the presence of various RSR' patterns (QRS < 120 ms) with or without Q wave which included an additional R wave (R prime) or the presence of more than 2 R primes in two contiguous leads. Notching was defined as notching of the S wave or the R wave without a distinct negative deflection within the R wave. Any QRS morphology with QRS >120 ms, including bundle branch block and intraventricular conduction delays, was defined as wide QRS. Follow-up data were collected from ICD recordings and included appropriate ICD shock and ATP as representation of VA occurrence, and inappropriate device therapy, the dates of their first occurrence and the date of last follow-up being registered as well. Appropriate device therapy was defined as the occurrence of a shock or ATP triggered by VF or sustained VT. An ICD shock or ATP occurring in the presence of atrial fibrillation, T-wave oversensing, regular supraventricular tachycardia, or noise was registered as inappropriate therapy. The number of non-sustained VTs and the cycle lengths of all VTs were also obtained. Furthermore, data on all-cause and cardiac mortality, VT ablation during follow-up, and complications, such as infection, perioperative complications, lead failure, and device failure were collected.

In general, ICDs implanted for primary prevention were programmed with a monitor zone from 150 bpm and a therapy zone from 200 bpm with long detection with a single shot ATP followed by maximal output shock therapy. In general, detection was programmed to 30 intervals for Medtronic, Abbott, or Biotronik devices. For Boston Scientific devices detection was set to 8/10 number of intervals to detect (NID) with a duration of 5 s leading to a total detection time of about 7 s (8 × 300 ms = 2.4 s and 2.4 s + 5 s = 7.4 s). Implantable cardioverter-defibrillators for secondary prevention were programmed with an additional VT therapy zone starting with a cycle length 20 ms less than the slowest documented VT and 2 burst ATPs followed by maximal output shock therapy. If available, morphology discrimination was used in the VT zone to reduce inappropriate shocks for SVT.

All ICD follow-up data, as well as the ECG data, were analyzed and validated by two cardiologists.

### LGE-CMR Analysis

**Figure 2:**
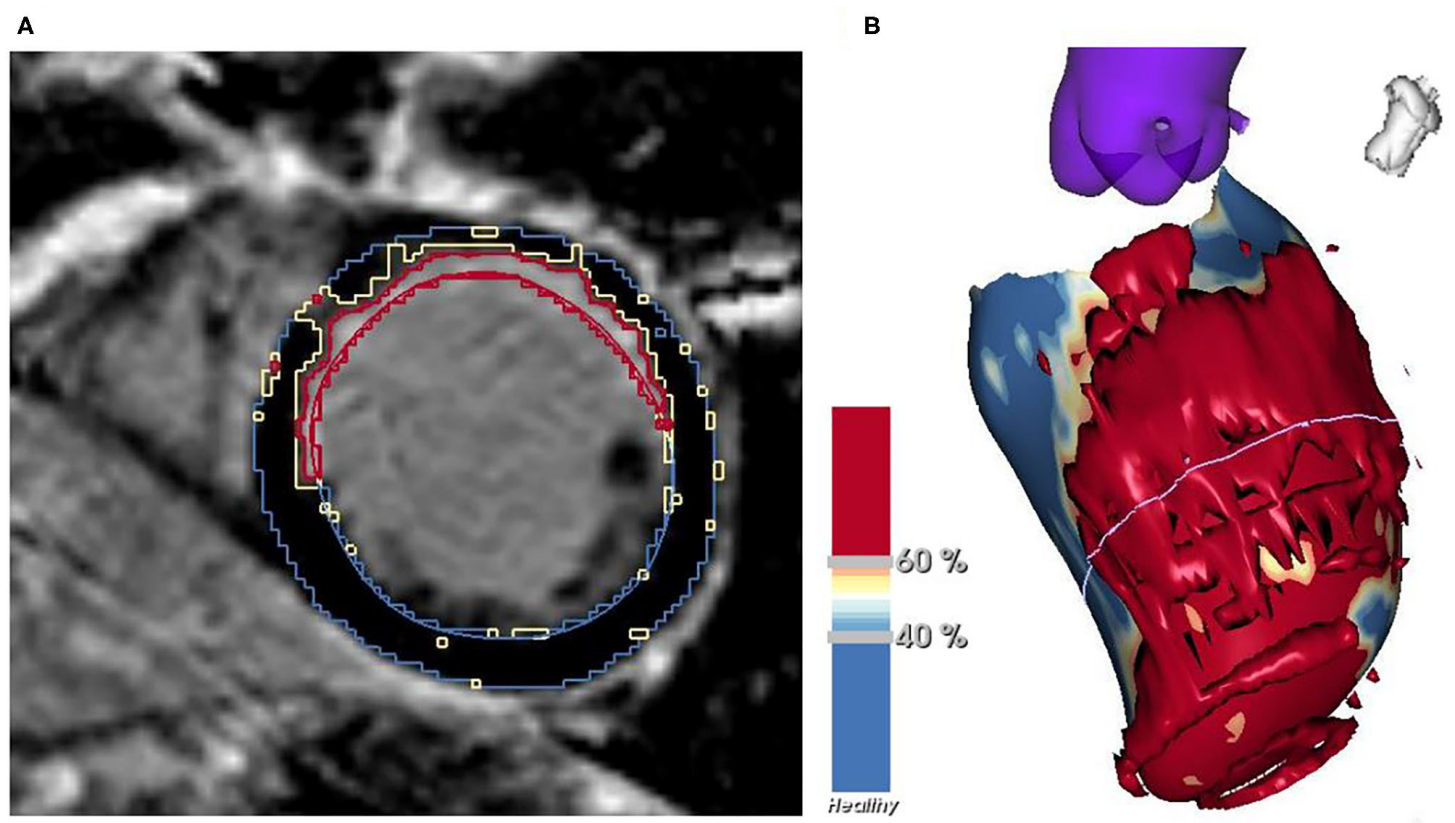
Myocardial scar characterization in a short-axis slice of LGE-CMR **(A)** and the resulting 3D model of the LV **(B)** with normal myocardium shown in blue, BZ in yellow and core in red. BZ, border zone; LGE-CMR, late gadolinium enhancement cardiac magnetic resonance; LV, left ventricle.

LGE-CMR images were obtained from all patients. Myocardial scar characterization was performed using 2D phase-sensitive inversion recovery (PSIR) images. An investigational software tool (ADAS 3D, Galgo Medical SL, Barcelona, Spain) was used to create a 3-dimensional (3D) model of the left ventricle (LV). After first placing several cardiac landmarks, a manual delineation of the endocardial and epicardial borders of the LV was performed in all short-axis slices with the use of a semiautomatic segmentation algorithm. The myocardial scar tissue was subdivided into two parts, namely core and BZ. The thresholds differentiating between core and BZ and between BZ and normal, healthy myocardium were determined for each patient separately on the basis of maximum pixel signal intensity (MPSI) with normal, healthy myocardium and infarct core serving as reference (Figure 2). BZ was thus identified as the transition zone between normal myocardium and infarct core. Optimal thresholds were determined based on their ability to correctly identify each of these areas. Quantitative data were obtained and included LV mass, total scar mass and percentage of the LV, BZ mass and percentage of the LV, and core mass and percentage of the LV.

In case of severe slice shifting, which refers to the displacement of slices with respect to each other, or if the contours of the heart were unclear, or if slices of a portion of the heart were absent, thus hindering the construction of a good 3D model of the LV, the LGE-CMR images were considered unsuitable for further analysis, which led to the exclusion of the patient concerned.

### Endpoints

The primary endpoint of this study was the occurrence of appropriate ICD therapy, which included shock and ATP and represents VA occurrence. Appropriate ICD shock was defined as a secondary endpoint. The composite of appropriate device therapy and cardiac mortality was equivalent to the primary endpoint.

### Statistical Analysis

A *t*-test for independent groups, Mann-Whitney test, and chi-square or Fisher's exact test were performed to compare patients with and without appropriate ICD therapy.

Cox proportional hazard regression analyses were performed to determine which myocardial scar characteristics, as obtained from LGE-CMR and ECG, are associated with the occurrence of appropriate ICD therapy and therefore also VA occurrence, after correcting for potential confounders. This was also done for the secondary endpoint. Univariable Cox regression analyses were performed for all variables stated in Supplementary Table 1 for both the primary and secondary endpoints. Variables with a *P* < 0.05 in univariable analysis (time since first myocardial infarction and digoxin at baseline for appropriate ICD therapy) and those clinically relevant (QRS fragmentation and indication for ICD placement) were selected for the multivariable analyses. Schoenfeld residuals were used to assess proportionality of hazards. Multivariable analyses were performed separately for all myocardial scar variables, because a strong correlation existed between them (Spearman's correlation coefficient 0.707; *P* < 0.001 for total scar mass and BZ mass). Variables that demonstrated multicollinearity (Spearman's correlation coefficient >0.7) with myocardial scar variables were excluded from the multivariable models. For the multivariable Cox regression analyses, missing data (time since first myocardial infarction) was dealt with by applying multiple imputation under the missing at random assumption. Forty imputed datasets were created using the predictive mean matching approach. Harrell's C-index was obtained for all Cox regression models to assess their goodness of fit. Kaplan-Meier curves were also constructed for the primary and secondary endpoints and were stratified by total scar mass and BZ mass. For this, the study population was split into two groups, the cut-off point being the median of the respective myocardial scar characteristics. The log-rank test was used to assess differences in cumulative event-free survival.

Statistical significance was defined by a *P* < 0.05. SPSS, version 23.0 (SPSS Institute, Chicago, IL, USA) and Stata 16 were used for statistical analyses.

## Results

### Baseline Characteristics

The study population consisted of 82 patients. Median age was 64 years (IQR 57–70 years). Seventy-four (90.2%) patients were men. The median time since the first myocardial infarction was 2.94 years (IQR 0.52–13.56 years). Patients with appropriate ICD therapy had a significantly larger time since the first myocardial infarction than those without appropriate ICD therapy [11.37 years (IQR 1.43–21.50 years) vs. 1.37 years (IQR 0.49–10.96 years); *P* = 0.034]. The QRS complex was normal in 15 (18.3%) patients, broad in 31 (37.8%) patients, notched in 24 (29.3%) patients and fragmented in 12 (14.6%) patients (Table 1).

**Table 1:** Baseline characteristics.

**Variable**	**Total study population**	**Appropriate ICD therapy**	**No appropriate ICD therapy**	***P*** **-value**
	**(***n*** = 82)**	**(***n*** = 24)**	**(***n*** = 58)**	
Age (years)	64 (57–70)	68 (59–72)	64 (56–70)	0.260
Male gender	74 (90.2%)	23 (95.8%)	51 (87.9%)	0.426
Indication ICD placement				0.084
Primary prevention	36 (43.9%)	7 (29.2%)	29 (50.0%)	
Secondary prevention	46 (56.1%)	17 (70.8%)	29 (50.0%)	
History of VAs				
VF	33 (40.2%)	12 (50.0%)	21 (36.2%)	0.222^a^
Sustained VT	13 (15.9%)	5 (20.8%)	8 (13.8%)	
Non-sustained VT	20 (24.4%)	10 (41.7%)	10 (17.2%)	0.019
Time since first MI (years)	2.94 (0.52–13.56)	11.37 (1.43–21.50)	1.37 (0.49–10.96)	0.034
NYHA class				1.000
I	33 (40.2%)	10 (41.7%)	23 (39.7%)	
II	39 (47.6%)	11 (45.8%)	28 (48.3%)	
III	9 (11.0%)	3 (12.5%)	6 (10.3%)	
IV	1 (1.2%)	0 (0.0%)	1 (1.7%)	
Medical history				
Atrial fibrillation				0.905
Paroxysmal	24 (29.3%)	6 (25.0%)	18 (31.0%)	
Permanent	3 (3.7%)	1 (4.2%)	2 (3.4%)	
MRI				
LVEF (%)	35 ± 12	35 ± 12	35 ± 13	0.973
ECG				
QRS complex				0.986
Normal	15 (18.3%)	4 (16.7%)	11 (19.0%)	
Broad (>120 ms)	31 (37.8%)	10 (41.7%)	21 (36.2%)	
Notching	24 (29.3%)	7 (29.2%)	17 (29.3%)	
Fragmentation	12 (14.6%)	3 (12.5%)	9 (15.5%)	
Laboratory measurements				
eGFR (ml/min/1.73 m^2^)	72 (60–86)	73 (60–84)	72 (60–89)	0.596
Medication				
Baseline				
ACE-I/ARB	60 (73.2%)	16 (66.7%)	44 (75.9%)	0.393
β-blocker	55 (67.1%)	16 (66.7%)	39 (67.2%)	0.960
Calcium antagonist	14 (17.1%)	5 (20.8%)	9 (15.5%)	0.538
Diuretic	35 (42.7%)	10 (41.7%)	25 (43.1%)	0.905
Statin	56 (68.3%)	16 (66.7%)	40 (69.0%)	0.839
MRA	21 (25.6%)	4 (16.7%)	17 (29.3%)	0.233
Antiarrhythmics				
Sotalol	2 (2.4%)	0 (0.0%)	2 (3.4%)	1.000
Amiodarone	3 (3.7%)	0 (0.0%)	3 (5.2%)	0.552
Digoxin	4 (4.9%)	3 (12.5%)	1 (1.7%)	0.073

*Categorical data are expressed as n (%) and continuous data as mean ± standard deviation (SD) or median and interquartile range (IQR) in case of a normal or skewed distribution, respectively. The P-values presented reflect a comparison between patients with and without appropriate ICD therapy. ACE-I, angiotensin-converting enzyme inhibitor; ARB, angiotensin receptor blocker; ECG, electrocardiography; eGFR, estimated glomerular filtration rate; ICD, implantable cardioverter-defibrillator; LVEF, left ventricular ejection fraction; MI, myocardial infarction; MRA, mineralocorticoid receptor antagonist; MRI, magnetic resonance imaging; NYHA, New York Heart Association; VA, ventricular arrhythmia; VF, ventricular fibrillation; VT, ventricular tachycardia*.

a *P-value for the combination of VF and sustained VT*.

### Events During Follow-Up

During a median duration of follow-up of 3.98 years (IQR 2.89–5.14 years), appropriate ICD therapy occurred 180 times in 24 (29.3%) patients. Seventy-four of these were appropriate shocks, which occurred in 19 (23.2%) patients. Eight (9.8%) patients died during follow-up, the cause of death being cardiac in 6 (7.3%) patients (Table 2).

**Table 2:** Events during follow-up.

**Outcomes**	**Total study population (*n* = 82)**
Appropriate ICD therapy	180 events in 24 (29.3%) patients^*^
Appropriate shock	74 events in 19 (23.2%) patients
Appropriate ATP	106 events in 20 (24.4%) patients
All-cause mortality	8 (9.8%)
Cardiac mortality	6 (7.3%)
Non-cardiac mortality	1 (1.2%)
Cause of death unknown	1 (1.2%)
Non-sustained VT	702 events in 29 (35.4%) patients
Cycle length of VTs (ms)	309 (250–350)
Inappropriate therapy	14 events in 5 (6.1%) patients
Lead failure	1 (1.2%)
Device failure	1 (1.2%)
Perioperative complications	1 (1.2%)
Infection	0 (0.0%)
VT ablation	4 (4.9%)

*Categorical data are expressed as n (%) and continuous data as median (IQR). ATP, antitachycardia pacing; ICD, implantable cardioverter-defibrillator; VT, ventricular tachycardia*.

* *Four therapies were given for VF, 4 for polymorphic VT and the remaining 172 events were monomorphic VT*.

### Myocardial Scar Characteristics

The median total scar mass of the study population was 49.0 g (IQR 34.3–64.7 g). The median BZ mass and core mass were 28.1 g (IQR 20.3–34.5 g) and 19.5 g (IQR 9.9–28.3 g), respectively. Patients who received appropriate ICD therapy had a significantly larger total scar mass than those who did not receive appropriate ICD therapy [60.0 g (IQR 41.2–73.4 g) vs. 43.3 g (IQR 31.2–61.2 g); *P* = 0.009]. Similarly, a significantly larger BZ mass was seen in patients with appropriate ICD therapy than in patients without appropriate ICD therapy [32.9 g (IQR 26.9–42.4 g) vs. 24.5 g (IQR 18.8–32.5 g); *P* = 0.001]. No significant difference in core mass was found between the two groups [21.8 g (IQR 13.3–29.8 g) vs. 17.6 g (IQR 7.9–28.5 g); *P* = 0.124] (Table 3).

**Table 3:** Myocardial scar characteristics.

**Variable**	**Total study population**	**Appropriate ICD therapy**	**No appropriate ICD therapy**	***P*** **-value**
	**(***n*** = 82)**	**(***n*** = 24)**	**(***n*** = 58)**	
LV mass (g)	183.2 ± 48.0	210.2 ± 50.1	172.0 ± 42.7	0.001
Total scar mass (g)	49.0 (34.3–64.7)	60.0 (41.2–73.4)	43.3 (31.2–61.2)	0.009
Total scar percentage of LV (%)	26.3 (20.5–35.3)	26.7 (24.5–35.0)	25.1 (18.8–36.5)	0.375
BZ mass (g)	28.1 (20.3–34.5)	32.9 (26.9–42.4)	24.5 (18.8–32.5)	0.001
BZ percentage of LV (%)	16.0 ± 4.3	17.4 ± 4.2	15.4 ± 4.3	0.065
Core mass (g)	19.5 (9.9–28.3)	21.8 (13.3–29.8)	17.6 (7.9–28.5)	0.124
Core percentage of LV (%)	10.1 (5.7–15.5)	10.0 (7.2–14.1)	10.2 (5.2–17.3)	0.647

*Data are expressed as mean ± SD or median (IQR). The P-values presented reflect a comparison between patients with and without appropriate ICD therapy. BZ, border zone; ICD, implantable cardioverter-defibrillator; LV, center ventricle; VA, ventricular arrhythmia*.

### Determinants of Appropriate ICD Therapy

**Figure 3:**
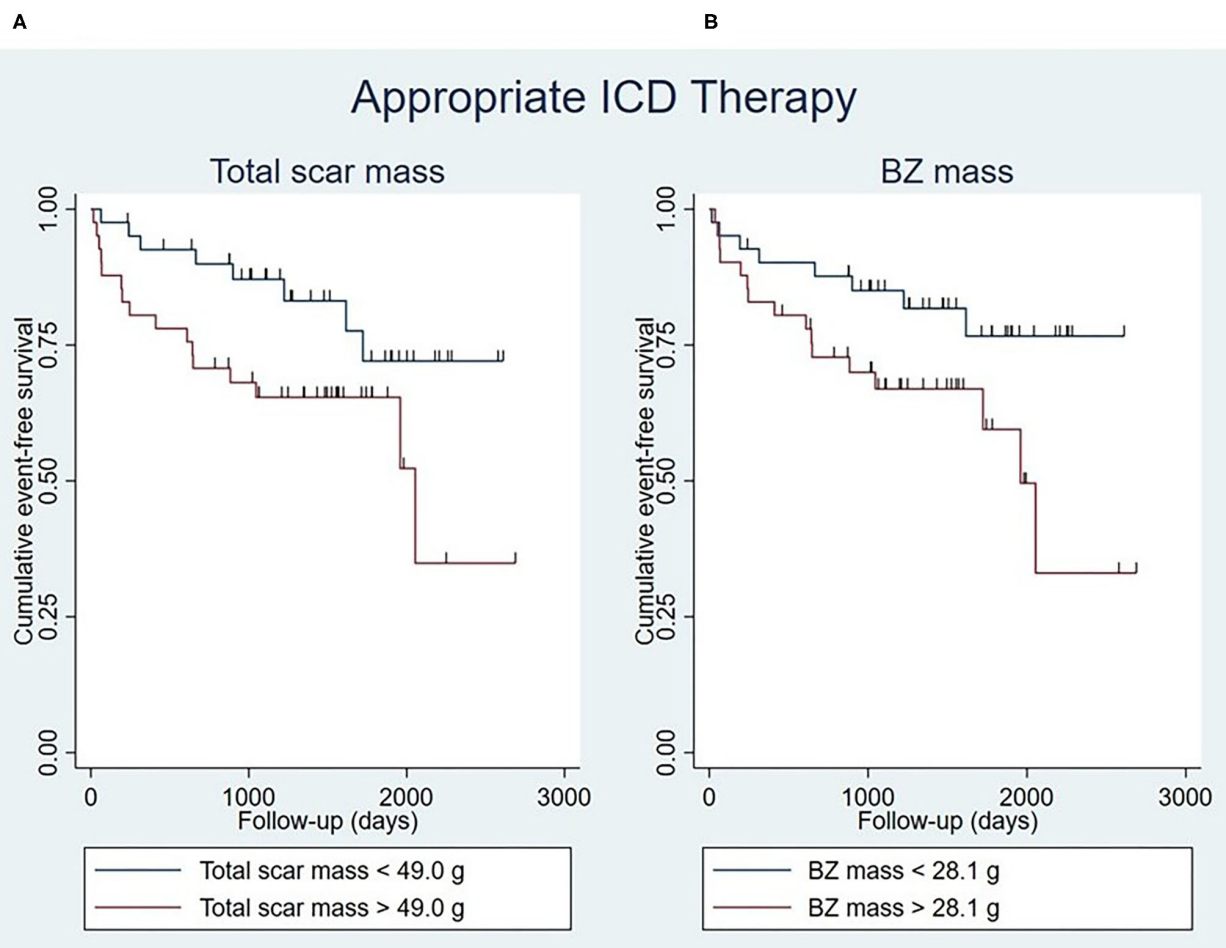
Kaplan-Meier curves for appropriate ICD therapy stratified by total scar mass (log-rank test *P* = 0.040) **(A)** and BZ mass (log-rank test *P* = 0.039) **(B)**, with the median of the respective myocardial scar characteristics being used as cut-off points. Censoring is indicated by vertical lines. BZ, border zone; ICD, implantable cardioverter-defibrillator.

Total scar mass was associated with the occurrence of appropriate ICD therapy, both univariably and multivariably [hazard ratio (HR) 1.02 [95% confidence interval (CI) 1.00–1.04]; *P* = 0.014] (Table 4). Patients with a large total scar mass (>49.0 g) were found to have lower event-free survival rates than those with a small total scar mass (<49.0 g) (*P* = 0.040), although a considerable number of events took place also in the latter group (Figure 3A).

**Table 4:** Cox regression analyses for the prediction of appropriate ICD therapy.

**Univariable analysis**			**Multivariable analysis** ^*^		
**Variable**	**HR (95% CI)**	***P*** **-value**	**Variable**	**HR (95% CI)**	***P*** **-value**
Total scar mass (g)	1.02 (1.00–1.04)	0.010	Total scar mass (g)	1.02 (1.00–1.04)	0.014
			Secondary prevention	2.16 (0.78–6.02)	0.140
			Time since first MI (years)	1.07 (1.00–1.14)	0.059
			Digoxin at baseline	5.21 (1.36–20.04)	0.016
			QRS complex		
			Broad (>120 ms)	0.85 (0.19–3.73)	0.829
			Notching	1.87 (0.49–7.07)	0.358
			Fragmentation	0.51 (0.09–2.80)	0.441
BZ mass (g)	1.04 (1.02–1.07)	0.001	BZ mass (g)	1.04 (1.01–1.07)	0.009
			Secondary prevention	1.99 (0.70–5.63)	0.195
			Time since first MI (years)	1.06 (0.99–1.13)	0.116
			Digoxin at baseline	5.52 (1.40–21.80)	0.015
			QRS complex		
			Broad (>120 ms)	0.78 (0.18–3.49)	0.746
			Notching	1.60 (0.41–6.16)	0.497
			Fragmentation	0.42 (0.07–2.47)	0.340
Core mass (g)	1.01 (0.99–1.04)	0.318	Core mass (g)	1.02 (0.99–1.04)	0.212
			Secondary prevention	2.25 (0.82–6.17)	0.114
			Time since first MI (years)	1.07 (1.00–1.14)	0.037
			Digoxin at baseline	5.02 (1.31–19.24)	0.019
			QRS complex		
			Broad (>120 ms)	1.03 (0.24–4.36)	0.971
			Notching	2.15 (0.56–8.32)	0.265
			Fragmentation	0.72 (0.13–3.82)	0.696

*The following are the reference categories of the categorical variables presented: primary prevention for ICD indication (presented in the table as secondary prevention) and normal for QRS complex. BZ, border zone; CI, confidence interval; HR, hazard ratio; ICD, implantable cardioverter-defibrillator; MI, myocardial infarction*.

* *LV mass was not corrected for due to the presence of multicollinearity (Spearman's correlation coefficient 0.752; P < 0.001 for LV mass and BZ mass)*.

Similar results were obtained for BZ mass, which was found to be a determinant of appropriate ICD therapy in univariable and multivariable analysis (HR 1.04 [95% CI 1.01–1.07]; *P* = 0.009) (Table 4). It was found that patients with a large BZ mass (>28.1 g) had lower event-free survival rates than patients with a small BZ mass (<28.1 g) (*P* = 0.039), although a considerable number of events took place also in the latter group (Figure 3B).

Core mass was not significantly associated with appropriate ICD therapy [HR 1.02 [95% CI 0.99–1.04]; *P* = 0.212 (multivariable analysis)] (Table 4).

The QRS complex was, when multivariably adjusted, not associated with the primary endpoint in any of the multivariable models. The time since the first myocardial infarction, however, was found to be an independent predictor of appropriate device therapy in four out of six multivariable models [e.g., HR 1.07 [95% CI 1.00–1.14]; *P* = 0.037 (multivariable model containing core mass)]. In five out of six multivariable models, digoxin usage at baseline was significantly associated with appropriate ICD therapy [e.g., HR 5.52 [95% CI 1.40–21.80]; *P* = 0.015 (multivariable model containing BZ mass)] (Table 4 and Supplementary Table 2).

The median Harrell's C-index of the multivariable models was 0.768 for total scar mass and 0.759 for BZ mass (Supplementary Table 3).

### Determinants of Appropriate ICD Shock

**Figure 4:**
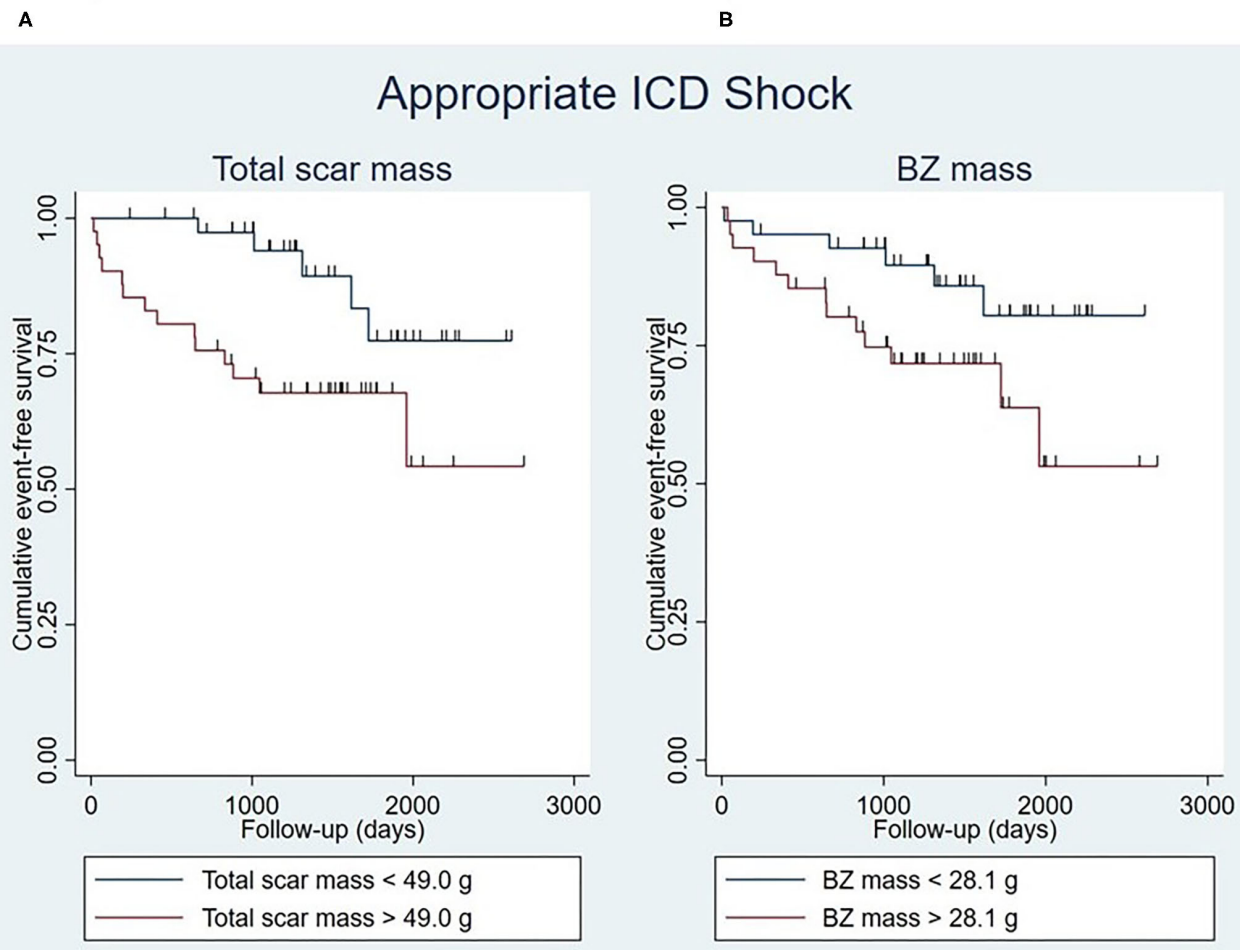
Kaplan-Meier curves for appropriate ICD shock stratified by total scar mass (log-rank test *P* = 0.016) **(A)** and BZ mass (log-rank test *P* = 0.054) **(B)**, with the median of the respective myocardial scar characteristics being used as cut-off points. Censoring is indicated by vertical lines. BZ, border zone; ICD, implantable cardioverter-defibrillator.

Total scar mass was significantly associated with appropriate ICD shock in univariable and multivariable analysis (Supplementary Table 4). Event-free survival rates were significantly lower in patients with a large total scar mass (>49.0 g) than in those whose total scar mass was small (<49.0 g) (*P* = 0.016) (Figure 4A).

Similarly, BZ mass was significantly associated with appropriate shock, both in univariable and multivariable analysis (Supplementary Table 4). Patients whose BZ mass was large (>28.1 g) seemed to have lower event-free survival rates than patients with a small BZ mass (<28.1 g), although this was not statistically significant (*P* = 0.054) (Figure 4B).

The median Harrell's C-index of the multivariable models was 0.810 for total scar mass and 0.817 for BZ mass (Supplementary Table 5).

## Discussion

This retrospective study aimed to investigate whether myocardial scar characterization improves risk stratification for VA occurrence in patients with ischemic cardiomyopathy who received an ICD. Total scar mass and BZ mass were associated with both appropriate ICD therapy and shock, although these myocardial scar characteristics seemed to be better at predicting appropriate shock than appropriate therapy. Core mass and the QRS complex, on the other hand, were not significantly associated with either endpoint. However, the time since the first myocardial infarction was significantly associated with appropriate device therapy in the majority of the Cox regression models.

Total scar mass and BZ mass were significantly associated with both appropriate ICD therapy and shock. This is consistent with previous findings (9–14). According to a meta-analysis by Disertori et al., the extents of total scar and BZ frequently predicted arrhythmic endpoints (18). This can be explained by the fact that myocardial scar tissue constitutes a substrate for VAs by causing re-entry (19). This is because the electrophysiological properties of the myocardium are disrupted by the presence of fibrosis (20). The predictive power of total scar mass, which is composed of BZ and core, is probably mainly due to the BZ component. The BZ of a myocardial scar consists of both fibrotic scar tissue and viable cardiomyocytes (20). It promotes slow conduction and can lead to re-entry in the following manner (20, 21): when two areas of normal myocardial tissue are connected by a channel of BZ tissue bordered on either side by scar core tissue, a re-entrant circuit can form (9). This makes the BZ a substrate for VAs (20), and explains its significant association with both endpoints. In general, the myocardial scar percentage variables were not significantly associated with appropriate therapy or shock, with the exception of BZ percentage of the LV, which was significantly associated with the latter. Although previous studies demonstrated a predictive value of such percentage variables (10, 12), our study showed that the absolute scar mass is of greater importance than the scar mass relative to the LV.

Core mass was not significantly associated with either endpoint, which is in line with findings from previous studies (12–14). Since the core of a myocardial scar contains no viable cardiomyocytes and therefore cannot participate in the re-entry phenomenon characteristic of VAs (11), it is only logical that the core mass was not significantly associated with appropriate device therapy or shock.

The QRS complex was not significantly associated with either endpoint. This is in contrast with the findings of a previous study, in which a fragmented QRS complex was found to be a predictor of arrhythmic events (22). Furthermore, since QRS fragmentation indicates the presence of a myocardial scar (22), we hypothesized that the former would be significantly associated with appropriate ICD therapy and shock. However, unlike LGE-CMR, the QRS complex was not able to stratify patients based on their risk in this study population.

The time since the first myocardial infarction was significantly associated with appropriate therapy in the majority of the Cox regression models, with older scars being more likely to lead to appropriate ICD therapy. This is in agreement with the findings of Piccini et al., who found a higher frequency of appropriate shocks when the time after myocardial infarction increased, although such a difference in frequency was not found for all-cause mortality or SCD (23). A potential explanation for this finding is that a post-infarction myocardial scar is not static. On the contrary, it is a dynamic tissue, undergoing continuous remodeling. In fact, myofibroblasts remain active in the scar tissue for years (24, 25). Perhaps this affects the arrhythmogenicity of the scar tissue such that older scars are more prone to trigger VAs. However, recent findings indicate that, in fact, the size of myocardial scars and the number of BZ channels decrease with time after an acute myocardial infarction (26). This is something that should be investigated further in future studies.

Digoxin usage at baseline was found to have predictive value in the majority of the multivariable models. This is in agreement with previous findings that digoxin usage is associated with a higher risk of appropriate shocks (27).

Based on Harrell's C-index, the myocardial scar characteristics of predictive significance seemed to be better at predicting appropriate ICD shock than appropriate therapy, the latter including both shock and ATP. This has beneficial clinical implications for several reasons. First, ATP usually occurs in the presence of relatively slow and hemodynamically stable VTs, whereas shocks are given under more severe circumstances, namely VF and relatively fast and hemodynamically unstable VTs (28). Second, while ATP does not cause any pain, shocks are painful (28). This makes patients receiving shocks more likely to benefit from antiarrhythmic drugs or catheter ablation than those receiving ATP.

Our findings may have clinical implications for patients. For instance, the use of total scar mass and BZ mass as risk stratification tools may help physicians to better select candidates for ICD implantation, since high-risk patients are in greater need of an ICD. However, there is a substantial burden associated with appropriate ICD therapies (5, 6). Antiarrhythmic drugs and catheter ablation are essential to reduce this burden (6). For example, the occurrence of VAs and consequently appropriate ICD therapies can be prevented by interrupting re-entry circuits through catheter ablation (24). Since nearly all ICD therapies in this study were given for monomorphic VTs rather than for VF, VT ablation constitutes a promising therapeutic option for patients similar to those in this study. However, determining which patients are likely to benefit from catheter ablation or antiarrhythmic drugs can be challenging. Currently, it is recommended to consider catheter ablation in patients with ischemic cardiomyopathy who received an ICD after the first occurrence of VT (17, 29, 30). Sapp et al. have shown that VT ablation is better for patients suffering from recurrent VT than escalation of antiarrhythmic drugs (31). However, results from previous studies among secondary prevention ICD recipients indicate that earlier prophylactic VT ablation around the time of ICD placement is associated with a reduction in the incidence of appropriate ICD therapy (32, 33). Knowledge of the total scar mass and BZ mass of patients, as obtained from LGE-CMR, may aid physicians in estimating the VA risk of their patients. This may help in deciding whether to perform catheter ablation or not, without having to wait until after the first occurrence of VT. The same can be applied to antiarrhythmic drugs. Thus, LGE-CMR-based, but not ECG-based myocardial scar characterization may be useful in clinical practice as a VA risk estimation tool to aid in the clinical decision making process. Ultimately, this could result in a reduction in the number of VTs as well as the negative effects associated with ICD therapies (6, 29). However, prior to implementation in clinical practice, more research is needed to validate our findings in a larger study population. Furthermore, future studies are required to investigate whether the use of myocardial scar characteristics in deciding which patients should be treated with antiarrhythmic drugs or catheter ablation leads to a reduction in VAs and consequently appropriate ICD therapies.

Our study investigated the anatomical substrate of VAs by LGE-CMR. However, it is worth mentioning that LGE-CMR cannot detect the neuronal changes that arise after the occurrence of a myocardial infarction. Innervation abnormalities are associated with a higher risk of VAs and are thus of clinical importance. However, the scar as defined by areas of reduced voltage does not adequately reflect the size of denervated areas, which has been shown to be 2.5 times larger (34). Knowledge of innervation abnormalities may help to improve outcomes of VT ablation. In addition, cardiac sympathetic denervation has been shown to be able to reduce VT burden (35). Therefore, this is an area that deserves further exploration in future studies.

To our knowledge, this study had one of the longest follow-up periods when compared to similar studies. However, there are several limitations that should be acknowledged. For instance, the relatively small sample size constitutes an important limitation. Furthermore, thresholds for the differentiation between normal myocardium, BZ, and core were not uniform for all patients, since this led to erroneous estimations of myocardial scar characteristics. Potential confounders were corrected for in the multivariable Cox regression models.

In conclusion, LGE-CMR-based, but not ECG-based myocardial scar characterization improves risk stratification for VA occurrence in patients with ischemic cardiomyopathy who received an ICD. More specifically, total scar mass and BZ mass were independent predictors of appropriate ICD therapy and shock, whereas core mass was not.

## Data Availability Statement

The raw data supporting the conclusions of this article will be made available by the authors, without undue reservation.

## Ethics Statement

Ethical review and approval was not required for the study on human participants in accordance with the local legislation and institutional requirements. Written informed consent for participation was not required for this study in accordance with the national legislation and the institutional requirements.

## Author Contributions

AN collected the data, performed the statistical analysis, and drafted the manuscript. AM collected data and drafted the manuscript. HG and BM provided critical review of the manuscript. MR performed statistical analysis and provided critical review of the manuscript and YB drafted the manuscript. All authors contributed to the article and approved the submitted version.

## Conflict of Interest

The authors declare that the research was conducted in the absence of any commercial or financial relationships that could be construed as a potential conflict of interest.

## Publisher's Note

All claims expressed in this article are solely those of the authors and do not necessarily represent those of their affiliated organizations, or those of the publisher, the editors and the reviewers. Any product that may be evaluated in this article, or claim that may be made by its manufacturer, is not guaranteed or endorsed by the publisher.
