# Supplementary material for: Myocardial Scar Characterization and Future Ventricular Arrhythmia in Patients With Ischemic Cardiomyopathy and an Implantable Cardioverter-Defibrillator
Source: Front Cardiovasc Med. 2021 Aug 17;8:708406. doi: 10.3389/fcvm.2021.708406 (PMC8415981; doi:10.3389/fcvm.2021.708406)
Supplement: Supplementary file 1 [file Data_Sheet_1.docx]

| **Supplementary Table 1. Baseline characteristics** | | | | |
| --- | --- | --- | --- | --- |
| **Variable** | **Total study population**  **(*n* = 82)** | **Appropriate ICD therapy**  **(*n* = 24)** | **No appropriate ICD therapy**  **(*n* = 58)** | ***P*-value** |
| Age (years)  Male gender  Indication ICD placement  Primary prevention  Secondary prevention  History of VAs  VF  Sustained VT  Non-sustained VT  ICD type  Subcutaneous  Transvenous  1 chamber  2 chambers  3 chambers  Time since first MI (years)  BMI (kg/m^2^)  NYHA class  I  II  III  IV  Medical history  Hypertension  Dyslipidemia  Diabetes  Atrial fibrillation  Paroxysmal  Permanent  Syncope  Coronary artery disease  Not proven*  1-vessel disease  2-vessel disease  3-vessel disease  MRI  LVEF (%)  LVEDV (mL)  LVESV (mL)  Right ventricular  functionº  Poor  Mediocre  Reasonable  Good  ECG  Ventricular heart rate  (bpm)  QRS complex§  Normal  Broad (>120ms)  Notching  Fragmentation  QRS morphology  Normal  LBBB  LAFB  RBBB  IVCD  LAFB+RBBB  QRS duration (ms)  QRS axis (degrees)  Early repolarization  Laboratory measurements  Hemoglobin (mmol/L)  Serum sodium  (mmol/L)  Serum potassium  (mmol/L)  Serum creatinine  (µmol/L)  eGFR (mL/min/1.73m^2^)  NT-proBNP (ng/L)  Medication  Baseline  ACE-I/ARB  β-blocker  Calcium antagonist  Diuretic  Statin  MRA  Antiarrhythmics  Sotalol  Amiodarone  Nitrate  Digoxin  Sacubitril/valsartan  Follow-up  ACE-I/ARB  β-blocker  Calcium antagonist  Diuretic  Statin  MRA  Antiarrhythmics  Sotalol  Amiodarone  Nitrate  Digoxin  Sacubitril/valsartan | 64 (57 – 70)  74 (90.2%)  36 (43.9%)  46 (56.1%)  33 (40.2%)  13 (15.9%)  20 (24.4%)  2 (2.4%)  50 (61.0%)  16 (19.5%)  14 (17.1%)  2.94 (0.52 – 13.56)  28.0 ± 4.2  33 (40.2%)  39 (47.6%)  9 (11.0%)  1 (1.2%)  46 (61.3%)  44 (55.7%)  20 (24.4%)  24 (29.3%)  3 (3.7%)  11 (13.4%)  5 (6.1%)  31 (37.8%)  22 (26.8%)  24 (29.3%)  35 ± 12  259 (216 – 307)  174 (124 – 213)  2 (2.5%)  9 (11.1%)  8 (9.9%)  62 (76.5%)  68 ± 14  15 (18.3%)  31 (37.8%)  24 (29.3%)  12 (14.6%)  25 (30.5%)  12 (14.6%)  14 (17.1%)  14 (17.1%)  14 (17.1%)  3 (3.7%)  111 (100 – 138)  21 (-14 – 64)  3 (3.7%)  8.7 (7.7 – 9.0)  139 (138 – 141)  4.3 ± 0.4  94 (80 – 109)  72 (60 – 86)  937 (348 – 1,766)  60 (73.2%)  55 (67.1%)  14 (17.1%)  35 (42.7%)  56 (68.3%)  21 (25.6%)  2 (2.4%)  3 (3.7%)  4 (4.9%)  4 (4.9%)  1 (1.2%)  78 (95.1%)  77 (93.9%)  21 (25.6%)  52 (63.4%)  74 (90.2%)  51 (62.2%)  6 (7.3%)  14 (17.1%)  9 (11.0%)  3 (3.7%)  13 (15.9%) | 68 (59 – 72)  23 (95.8%)  7 (29.2%)  17 (70.8%)  12 (50.0%)  5 (20.8%)  10 (41.7%)  0 (0.0%)  14 (58.3%)  7 (29.2%)  3 (12.5%)  11.37 (1.43 – 21.50)  28.1 ± 2.7  10 (41.7%)  11 (45.8%)  3 (12.5%)  0 (0.0%)  13 (59.1%)  14 (63.6%)  8 (33.3%)  6 (25.0%)  1 (4.2%)  4 (16.7%)  2 (8.3%)  8 (33.3%)  7 (29.2%)  7 (29.2%)  35 ± 12  253 (223 – 310)  171 (134 – 248)  1 (4.2%)  3 (12.5%)  3 (12.5%)  17 (70.8%)  68 ± 16  4 (16.7%)  10 (41.7%)  7 (29.2%)  3 (12.5%)  7 (29.2%)  2 (8.3%)  1 (4.2%)  4 (16.7%)  7 (29.2%)  3 (12.5%)  115 (100 – 140)  45 (-12 – 67)  1 (4.2%)  8.4 (7.5 – 9.3)  140 (137 – 141)  4.4 ± 0.4  91 (86 – 108)  73 (60 – 84)  1,000 (560 – 1,439)  16 (66.7%)  16 (66.7%)  5 (20.8%)  10 (41.7%)  16 (66.7%)  4 (16.7%)  0 (0.0%)  0 (0.0%)  2 (8.3%)  3 (12.5%)  0 (0.0%)  23 (95.8%)  23 (95.8%)  5 (20.8%)  17 (70.8%)  21 (87.5%)  16 (66.7%)  3 (12.5%)  7 (29.2%)  2 (8.3%)  3 (12.5%)  4 (16.7%) | 64 (56 – 70)  51 (87.9%)  29 (50.0%)  29 (50.0%)  21 (36.2%)  8 (13.8%)  10 (17.2%)  2 (3.4%)  36 (62.1%)  9 (15.5%)  11 (19.0%)  1.37 (0.49 – 10.96)  28.0 ± 4.7  23 (39.7%)  28 (48.3%)  6 (10.3%)  1 (1.7%)  33 (62.3%)  30 (52.6%)  12 (20.7%)  18 (31.0%)  2 (3.4%)  7 (12.1%)  3 (5.2%)  23 (39.7%)  15 (25.9%)  17 (29.3%)  35 ± 13  259 (211 – 303)  175 (123 – 209)  1 (1.8%)  6 (10.5%)  5 (8.8%)  45 (78.9%)  68 ± 13  11 (19.0%)  21 (36.2%)  17 (29.3%)  9 (15.5%)  18 (31.0%)  10 (17.2%)  13 (22.4%)  10 (17.2%)  7 (12.1%)  0 (0.0%)  109 (99 – 138)  16 (-18 – 64)  2 (3.4%)  8.8 (7.7 – 9.0)  139 (138 – 141)  4.3 ± 0.3  95 (78 – 112)  72 (60 – 89)  784 (314 – 2,108)  44 (75.9%)  39 (67.2%)  9 (15.5%)  25 (43.1%)  40 (69.0%)  17 (29.3%)  2 (3.4%)  3 (5.2%)  2 (3.4%)  1 (1.7%)  1 (1.7%)  55 (94.8%)  54 (93.1%)  16 (27.6%)  35 (60.3%)  53 (91.4%)  35 (60.3%)  3 (5.2%)  7 (12.1%)  7 (12.1%)  0 (0.0%)  9 (15.5%) | 0.260  0.426  0.084  0.222^a^  0.019  0.476  0.034  0.847  1.000  0.797  0.377  0.225  0.905  0.723  0.900  0.973  0.763  0.654  0.733  0.976  0.986  0.016  0.456  0.415  1.000  0.574  0.988  0.308  0.596  0.596  0.616  0.393  0.960  0.538  0.905  0.839  0.233  1.000  0.552  0.577  0.073  1.000  1.000  1.000  0.524  0.370  0.687  0.591  0.352  0.103  1.000  0.023  1.000 |

Categorical data are expressed as *n* (%) and continuous data as mean ± standard deviation (SD) or median and interquartile range (IQR) in case of a normal or skewed distribution, respectively. The *P*-values presented reflect a comparison between patients with and without appropriate ICD therapy.

Abbreviations: ACE-I = angiotensin-converting enzyme inhibitor; ARB = angiotensin receptor blocker; BMI = body mass index; ECG = electrocardiography; eGFR = estimated glomerular filtration rate; ICD = implantable cardioverter-defibrillator; IVCD = intraventricular conduction delay; LAFB = left anterior fascicular block; LBBB = left bundle branch block; LVEDV = left ventricular end-diastolic volume; LVEF = left ventricular ejection fraction; LVESV = left ventricular end-systolic volume; MI = myocardial infarction; MRA = mineralocorticoid receptor antagonist; MRI = magnetic resonance imaging; NT-proBNP = N-terminal pro-brain natriuretic peptide; NYHA = New York Heart Association; RBBB = right bundle branch block; VA = ventricular arrhythmia; VF = ventricular fibrillation; VT = ventricular tachycardia.

* The presence of coronary artery disease required a stenosis of ≥70% as measured by coronary angiography.

º Right ventricular function was determined by two independent cardiologists based on imaging results from transthoracic ultrasound.

§ Fragmented QRS was defined by the presence of various RSR’ patterns (QRS<120ms) with or without Q wave which included an additional R wave (R prime) or the presence of more than 2 R primes in two contiguous leads. Notching was defined as notching of the S wave or the R wave without a distinct negative deflection within the R wave. Any QRS morphology with QRS >120ms, including bundle branch block and intraventricular conduction delays, was defined as wide QRS.

^a^ *P*-value for the combination of VF and sustained VT.

| **Supplementary Table 2. Cox regression analyses for the prediction of appropriate ICD therapy** | | | | | |
| --- | --- | --- | --- | --- | --- |
| **Univariable analysis** | | | **Multivariable analysis*** | | |
| **Variable** | **HR (95% CI)** | ***P*-value** | **Variable** | **HR (95% CI)** | ***P*-value** |
| Total scar mass (g) | 1.02 (1.00 – 1.04) | 0.010 | Total scar mass (g)  Secondary prevention  Time since first MI (years)  Digoxin at baseline  QRS complex  - Broad (>120ms)  - Notching  - Fragmentation | 1.02 (1.00 – 1.04)  2.16 (0.78 – 6.02)  1.07 (1.00 – 1.14)  5.21 (1.36 – 20.04)  0.85 (0.19 – 3.73)  1.87 (0.49 – 7.07)  0.51 (0.09 – 2.80) | 0.014  0.140  0.059  0.016  0.829  0.358  0.441 |
| Total scar percentage of LV (%) | 1.01 (0.97 – 1.04) | 0.727 | Total scar percentage of LV (%)  Secondary prevention  Time since first MI (years)  Digoxin at baseline  QRS complex  - Broad (>120ms)  - Notching  - Fragmentation | 1.02 (0.97 – 1.06)  2.23 (0.82 – 6.08)  1.07 (1.00 – 1.14)  4.46 (1.12 – 17.76)  1.07 (0.26 – 4.47)  2.09 (0.54 – 8.17)  0.73 (0.14 – 3.94) | 0.467  0.118  0.035  0.034  0.929  0.288  0.716 |
| BZ mass (g) | 1.04 (1.02 – 1.07) | 0.001 | BZ mass (g)  Secondary prevention  Time since first MI (years)  Digoxin at baseline  QRS complex  - Broad (>120ms)  - Notching  - Fragmentation | 1.04 (1.01 – 1.07)  1.99 (0.70 – 5.63)  1.06 (0.99 – 1.13)  5.52 (1.40 – 21.80)  0.78 (0.18 – 3.49)  1.60 (0.41 – 6.16)  0.42 (0.07 – 2.47) | 0.009  0.195  0.116  0.015  0.746  0.497  0.340 |
| BZ percentage of LV (%) | 1.09 (1.00 – 1.19) | 0.058 | BZ percentage of LV (%)  Secondary prevention  Time since first MI (years)  Digoxin at baseline  QRS complex  - Broad (>120ms)  - Notching  - Fragmentation | 1.08 (0.98 – 1.20)  2.08 (0.76 – 5.72)  1.07 (1.00 – 1.14)  3.43 (0.81 – 14.47)  0.99 (0.24 – 4.09)  1.70 (0.43 – 6.70)  0.60 (0.11 – 3.18) | 0.132  0.157  0.045  0.093  0.989  0.449  0.544 |
| Core mass (g) | 1.01 (0.99 – 1.04) | 0.318 | Core mass (g)  Secondary prevention  Time since first MI (years)  Digoxin at baseline  QRS complex  - Broad (>120ms)  - Notching  - Fragmentation | 1.02 (0.99 – 1.04)  2.25 (0.82 – 6.17)  1.07 (1.00 – 1.14)  5.02 (1.31 – 19.24)  1.03 (0.24 – 4.36)  2.15 (0.56 – 8.32)  0.72 (0.13 – 3.82) | 0.212  0.114  0.037  0.019  0.971  0.265  0.696 |
| Core percentage of LV (%) | 0.99 (0.95 – 1.03) | 0.679 | Core percentage of LV (%)  Secondary  prevention  Time since first MI (years)  Digoxin at baseline  QRS complex  - Broad (>120ms)  - Notching  - Fragmentation | 1.00 (0.95 – 1.06)  2.19 (0.80 – 5.97)  1.07 (1.00 – 1.14)  4.97 (1.28 – 19.31)  1.16 (0.27 – 4.90)  2.28 (0.58 – 8.97)  0.86 (0.16 – 4.57) | 0.933  0.128  0.043  0.021  0.840  0.237  0.858 |

The following are the reference categories of the categorical variables presented: primary prevention for ICD indication (presented in the table as secondary prevention) and normal for QRS complex.

Abbreviations: BZ = border zone; CI = confidence interval; HR = hazard ratio; ICD = implantable cardioverter-defibrillator; LV = left ventricle; MI = myocardial infarction.

* LV mass was not corrected for due to the presence of multicollinearity (Spearman’s correlation coefficient 0.752; *P*<0.001 for LV mass and BZ mass).

| **Supplementary Table 3. Harrell’s C-index for the Cox regression models - appropriate ICD therapy** | | |
| --- | --- | --- |
| **Variable** | **Univariable** | **Multivariable*** |
| Total scar mass (g) | 0.651 | 0.768 (0.727 – 0.814) |
| Total scar percentage of LV (%) | 0.566 | 0.755 (0.699 – 0.789) |
| BZ mass (g) | 0.667 | 0.759 (0.714 – 0.785) |
| BZ percentage of LV (%) | 0.599 | 0.752 (0.717 – 0.778) |
| Core mass (g) | 0.614 | 0.763 (0.711 – 0.792) |
| Core percentage of LV (%) | 0.437 | 0.749 (0.695 – 0.794) |

Abbreviations: BZ = border zone; ICD = implantable cardioverter-defibrillator; LV = left ventricle.

* Harrell’s C-index is presented as median (range) of the 40 imputed datasets.

| **Supplementary Table 4. Cox regression analyses for the prediction of** **appropriate ICD shock** | | | | | |
| --- | --- | --- | --- | --- | --- |
| **Univariable analysis** | | | **Multivariable analysis*** | | |
| **Variable** | **HR (95% CI)** | ***P*-value** | **Variable** | **HR (95% CI)** | ***P*-value** |
| Total scar mass (g) | 1.03 (1.01 – 1.04) | 0.005 | Total scar mass (g)  Secondary prevention  Time since first MI (years)  Digoxin at baseline  Serum sodium (mmol/L)  QRS complex  - Broad (>120ms)  - Notching  - Fragmentation | 1.03 (1.01 – 1.05)  4.15 (1.14 – 15.14)  1.04 (0.97 – 1.11)  5.26 (0.93 – 29.87)  0.95 (0.81 – 1.11)  1.41 (0.28 – 7.12)  2.23 (0.49 – 10.20)  1.17 (0.18 – 7.51) | 0.006  0.031  0.309  0.061  0.509  0.676  0.303  0.868 |
| Total scar percentage of LV (%) | 1.02 (0.98 – 1.06) | 0.314 | Total scar percentage of LV (%)  Secondary prevention  Time since first MI (years)  Digoxin at baseline  Serum sodium  (mmol/L)  QRS complex  - Broad (>120ms)  - Notching  - Fragmentation | 1.04 (0.99 – 1.09)  4.48 (1.24 – 16.12)  1.05 (0.99 – 1.12)  5.59 (1.01 – 30.89)  0.99 (0.85 – 1.16)  1.51 (0.33 – 6.82)  2.07 (0.46 – 9.42)  1.23 (0.21 – 7.35) | 0.166  0.022  0.124  0.049  0.919  0.592  0.345  0.820 |
| BZ mass (g) | 1.05 (1.02 – 1.08) | 0.001 | BZ mass (g)  Secondary prevention  Time since first MI (years)  Digoxin at baseline  Serum sodium (mmol/L)  QRS complex  - Broad (>120ms)  - Notching  - Fragmentation | 1.06 (1.02 – 1.10)  3.25 (0.87 – 12.11)  1.02 (0.95 – 1.10)  4.54 (0.74 – 27.96)  0.91 (0.77 – 1.09)  1.25 (0.25 – 6.31)  1.59 (0.35 – 7.26)  0.94 (0.14 – 6.20) | 0.005  0.079  0.616  0.103  0.314  0.784  0.548  0.948 |
| BZ percentage of LV (%) | 1.14 (1.03 – 1.26) | 0.011 | BZ percentage of LV (%)  Secondary prevention  Time since first MI (years)  Digoxin at baseline  Serum sodium (mmol/L)  QRS complex  - Broad (>120ms)  - Notching  - Fragmentation | 1.13 (1.01 – 1.26)  3.56 (0.98 – 12.94)  1.04 (0.97 – 1.11)  3.51 (0.54 – 22.81)  0.96 (0.82 – 1.13)  1.50 (0.33 – 6.82)  1.68 (0.37 – 7.65)  1.11 (0.19 – 6.55) | 0.034  0.054  0.236  0.188  0.657  0.603  0.505  0.908 |
| Core mass (g) | 1.02 (0.99 – 1.04) | 0.211 | Core mass (g)  Secondary prevention  Time since first MI (years)  Digoxin at baseline  Serum sodium (mmol/L)  QRS complex  - Broad (>120ms)  - Notching  - Fragmentation | 1.02 (0.99 – 1.05)  4.53 (1.25 – 16.47)  1.05 (0.98 – 1.12)  6.65 (1.24 – 35.78)  0.99 (0.85 – 1.15)  1.53 (0.33 – 7.07)  2.28 (0.50 – 10.45)  1.36 (0.23 – 8.17) | 0.121  0.022  0.144  0.027  0.883  0.585  0.287  0.735 |
| Core percentage of LV (%) | 1.00 (0.96 – 1.05) | 0.960 | Core percentage of LV (%)  Secondary prevention  Time since first MI (years)  Digoxin at baseline  Serum sodium (mmol/L)  QRS complex  - Broad (>120ms)  - Notching  - Fragmentation | 1.02 (0.96 – 1.07)  4.32 (1.19 – 15.67)  1.05 (0.98 – 1.12)  6.86 (1.28 – 36.92)  1.00 (0.85 – 1.16)  1.67 (0.37 – 7.59)  2.28 (0.49 – 10.55)  1.51 (0.26 – 8.97) | 0.603  0.026  0.142  0.025  0.950  0.507  0.292  0.648 |

The following are the reference categories of the categorical variables presented: primary prevention for ICD indication (presented in the table as secondary prevention) and normal for QRS complex.

Abbreviations: BZ = border zone; CI = confidence interval; HR = hazard ratio; ICD = implantable cardioverter-defibrillator; LV = left ventricle; MI = myocardial infarction.

* LV mass was not corrected for due to the presence of multicollinearity (Spearman’s correlation coefficient 0.752; *P*<0.001 for LV mass and BZ mass).

| **Supplementary Table 5. Harrell’s C-index for the Cox regression models - appropriate ICD shock** | | |
| --- | --- | --- |
| **Variable** | **Univariable** | **Multivariable*** |
| Total scar mass (g) | 0.697 | 0.810 (0.782 – 0.833) |
| Total scar percentage of LV (%) | 0.623 | 0.794 (0.747 – 0.831) |
| BZ mass (g) | 0.722 | 0.817 (0.797 – 0.839) |
| BZ percentage of LV (%) | 0.679 | 0.813 (0.793 – 0.843) |
| Core mass (g) | 0.641 | 0.783 (0.749 – 0.821) |
| Core percentage of LV (%) | 0.595 | 0.777 (0.734 – 0.814) |

Abbreviations: BZ = border zone; ICD = implantable cardioverter-defibrillator; LV = left ventricle.

* Harrell’s C-index is presented as median (range) of the 40 imputed datasets.
